# Impact of Etiology on Mortality and Recovery in Patients With Status Epilepticus

**DOI:** 10.1212/WNL.0000000000214624

**Published:** 2026-02-04

**Authors:** Pia De Stefano, Sira Maria Baumann, Urs Fisch, Pascale Susanne Grzonka, Tommaso Rochat, Gian Marco De Marchis, Tolga Daniel Dittrich, Sabina Hunziker, Stephan J. Rüegg, Andreas Kleinschmidt, Hervé Quintard, Margitta Seeck, Raoul Sutter

**Affiliations:** 1 Neuro-Intensive Care Unit, Department of Intensive Care, University Hospital of Geneva, Switzerland;; 2 Clinic for Intensive Care Medicine, University Hospital Basel, Switzerland;; 3 Department of Neurology, Brigham and Women's Hospital, Boston, MA;; 4 Department of Neurology, University Hospital Basel, Switzerland;; 5 Department of Neurology, University Teaching and Research Hospital, HOCH, Cantonal Hospital St. Gallen, Switzerland;; 6 Medical Faculty of the University of Basel, Switzerland;; 7 Medical Communication and Psychosomatic Medicine, University Hospital Basel, Switzerland;; 8 EEG & Epilepsy Unit, Department of Clinical Neurosciences, University Hospital of Geneva, Switzerland; and; 9 Medical Faculty of the University of Geneva, Switzerland.

## Abstract

**Background and Objectives:**

Although etiology is considered central to outcomes in status epilepticus (SE), previous studies often lacked standardized classification and adjustment for confounders, particularly withdrawal of life-sustaining treatment (WLST). This study examined the association between SE etiology, mortality, and neurologic recovery using the International League Against Epilepsy (ILAE) classification while accounting for confounders and WLST.

**Methods:**

This 2-center observational study included adults (≥18 years) with SE treated at the University Hospitals of Basel and Geneva from 2015 to 2023. Etiologies were classified as acute symptomatic, remote symptomatic-unprovoked, progressive CNS disorders, epilepsy without additional triggers, or cryptogenic. Demographics, SE type, SE severity score, Charlson Comorbidity Index, treatment data, complications, and WLST were assessed. The primary outcome was in-hospital mortality; secondary outcomes were 30-day mortality and recovery to premorbid neurologic function at discharge. Associations were assessed using Poisson regression with robust error variance, adjusted for age, nonconvulsive SE (NCSE) with coma, comorbidity, and center.

**Results:**

Among 967 patients (median age 67 years, interquartile range 54–78; 46.5% female), SE was terminated in 95%, with 48.5% of patients recovering to premorbid function. Acute symptomatic SE accounted for 34.2%, remote symptomatic SE for 27.6%, SE due to progressive CNS disorders for 14.4%, epilepsy without additional triggers for 16.7%, and cryptogenic SE for 7.1%. In-hospital and 30-day mortality were 7.9% and 13.9%, respectively, while 48.5% recovered to premorbid function. Etiology was associated with neurologic recovery, with intracranial hemorrhage (relative risk [RR] 0.49, 95% CI 0.35–0.67) and acute symptomatic SE (RR 0.71, 95% CI 0.60–0.83) being associated with reduced likelihood of recovery, whereas known epilepsy was associated with increased likelihood of recovery (RR 1.40, 95% CI 1.23–1.60). NCSE with coma (11.9%) was independently associated with higher in-hospital and 30-day mortality and reduced recovery across all ILAE etiology groups. WLST did not significantly alter these associations.

**Discussion:**

Etiology was associated with neurologic recovery but not with short-term mortality after adjustment for confounders and WLST. By contrast, NCSE with coma showed the strongest association with adverse outcomes. This suggests that while etiology informs prognosis for recovery, SE type, particularly NCSE with coma, is the more critical determinant of survival.

## Introduction

Status epilepticus (SE) is associated with significant morbidity and mortality,^1-5^ requiring rapid diagnosis and treatment to improve outcomes. SE etiology plays a critical role in prognosis, with studies suggesting that it is an important, if not the most important, determinant of survival and neurologic recovery.^1-3,5-9^ Despite these insights, sound conclusions and a high level of evidence could not be established because etiologic categorization was not standardized in early studies. This has motivated the International League Against Epilepsy (ILAE) to propose a categorization and classification of SE etiologies into specific etiology groups.^10,11^ Despite these efforts, studies applying the proposed etiologic categorization and adjusting for potential confounding factors remain scarce. These limitations are further compounded by challenges related to withdrawal of life-sustaining measures. Decisions regarding such withdrawal are influenced by various factors, such as adherence to patient directives or the determination that all therapeutic options have been exhausted in the context of a presumed poor prognosis.

Withdrawal of life-sustaining treatment (WLST) can influence mortality rates, potentially biasing the link between SE etiology and outcomes. To clarify this relationship, we evaluated whether etiologies, based on the ILAE classification,^10,11^ were associated with in-hospital and 30-day mortality, as well as neurologic recovery at discharge, after adjusting for confounders and accounting for life-sustaining treatment decisions in a large cohort.

## Methods

### Standard Protocol Approvals, Registrations, and Patient Consents

Patient consent was waived by the ethics committees (Ethikkommission Nordwest- und Zentralschweiz 2019-00693 and Commission Cantonale d'Ethique de la Recherche 2019-00836) following the Declaration of Helsinki from 1964 and amendments.

The study is reported using Strengthening the Reporting of Observational Studies in Epidemiology (STROBE) guidelines.^12^

### Data Collection

This Swiss observational 2-center study was conducted at the University Hospitals of Basel and Geneva, 2 tertiary care centers. The data of this study are part of the Swiss STatus Epilepticus Population Study (ClinicalTrials.gov NCT04204863). From January 1, 2015, to December 31, 2023, clinical, laboratory, and epileptologic data of all consecutive adult patients (i.e., ≥18 years) with SE were compiled. Data were collected from electronic records and entered into a password-protected Research Electronic Data Capture database.^13^

Collected variables included demographics and presumed SE etiology, subsequently categorized according to the ILAE^10,11^ as acute symptomatic, for example, stroke or encephalitis; remote symptomatic, for example, remote stroke or post-traumatic; progressive CNS disorder, for example, slow-growing brain tumors; SE in defined electroclinical syndromes, for example, juvenile myoclonic epilepsy or Lennox-Gastaut syndrome; or unprovoked/unknown (i.e., cryptogenic) cause. Of note, specific etiologies were non–mutually exclusive because some patients had multiple presumed etiologies. Depending on context, certain etiologies could belong to different ILAE categories (e.g., brain tumor–related seizures classified as seizures from progressive CNS disorders if slow-growing or acute symptomatic if rapidly growing). In cases of coexisting etiologies, classification followed physician judgment regarding the leading and ILAE-defining etiology. Type of SE was assessed according to the ILAE^11^ as SE with prominent motor symptoms (including convulsive and myoclonic), nonconvulsive SE (NCSE) with coma, and NCSE without coma (including generalized and focal). Because, in retrospect, an identification of specific electroclinical syndromes and their separation from patients experiencing SE because of underlying epilepsy without identifiable additional triggers was not possible, we defined a category including patients with SE from epilepsy without additional triggers and those with electroclinical syndromes. Patients with posthypoxic SE were not included, as explained elsewhere.^14^

Severity of illness was assessed using the Status Epilepticus Severity Score (STESS; range 0–6^15,16^), SE duration, and Charlson Comorbidity Index (CCI; range 0–37^17^).

At both centers, continuous EEG (cEEG) monitoring was performed with at least 21 electrodes. cEEG was performed in Basel during the management of treatment-refractory SE, if NCSE is suspected. In Geneva, spot EEG for 30 minutes or on-demand cEEG was performed from 2015 to 2019, to monitor SE. From 2020 onward, cEEG indications expanded to the management of refractory SE (RSE), suspected NCSE, and altered mental status with lack of clinical explanation.

Convulsive SE was defined as convulsive seizures lasting >5 minutes, whereas other SE types (including myoclonic and NCSE with or without coma) were defined after 10 minutes of ongoing seizures (electrographically, as defined by the Salzburg criteria or the American Clinical Neurophysiology Society standardized terminology, or clinically). On cEEG, SE was also diagnosed if recurrent seizures occupied >20% of 1-hour recordings.^11,18^

The duration was defined as the time from clinical/EEG evidence of seizure onset to EEG-confirmed seizure termination. Owing to the use of 2 EEG monitoring strategies (cEEG for several hours or spot EEGs for at least 20 minutes every 12 hours), SE duration was approximated with a potential inaccuracy of ≤12 hours.

For SE with prominent motor symptoms, SE onset was defined clinically, and for NCSE, SE onset was defined by EEG.^18^ With anesthesia, the end of SE was defined as the point when a seizure-free EEG pattern was achieved, and this state persisted after the withdrawal of anesthetic drugs. Refractory SE was defined according to the Neurocritical Care Society guidelines,^19^ requiring failure of both benzodiazepines (i.e., first-line) and nonbenzodiazepine antiseizure medications (i.e., second-line), in-hospital and intensive care unit (ICU) length of stay, duration of mechanical ventilation, use of nonsedating antiseizure medication, continuous anesthetic administration, and vasopressor support. Documented complications included infections, arterial hypotension, and organ failure.

Data on WLST were recorded, such as the time (during or after SE) and the reason (e.g., due to lack of treatment options, poor prognosis, or confirmed/presumed patient directives).

### Antiseizure Treatment

Throughout the study, treatment followed Neurocritical Care Society and the American Epilepsy Society guidelines under consistent neurologist and neurointensivist supervision.^19,20^ First-line therapy involved intravenous benzodiazepines, repeated if needed. For benzodiazepine-refractory SE, second-line treatment with levetiracetam, lacosamide, valproic acid, or phenytoin was initiated. In treatment-RSE, nonsedating antiseizure drugs (e.g., topiramate, zonisamide, oxcarbazepine, pregabalin, or perampanel) were added and continuous propofol or midazolam infusion was started. Anesthetics were titrated to termination of seizures or the establishment of burst suppression at the physician's discretion.^21-23^ If seizures recurred, barbiturates were usually administered to induce burst suppression.

### Outcomes

In-hospital mortality in relation to the etiology of SE and predefined etiology groups, classified according to the ILAE criteria,^10,11^ was the primary outcome. Secondary outcomes included 30-day mortality after SE and survival with a return to premorbid neurologic function at hospital discharge.

Return to premorbid neurologic function was defined as the full recovery of all patients' neurologic abilities, or restoration of neurologic functioning to the level present before SE, based on physicians' notes and examination.

### Statistical Analysis

Categorical data were expressed as frequencies and percentages while continuous data were presented as medians and interquartile ranges (IQRs). Participants were grouped into survivors and nonsurvivors. The Shapiro-Wilk test determined data normality. Continuous variables were compared using either the Student *t* test (normal distribution) or the Mann-Whitney *U* test (non-normal distribution). Proportional comparisons were made with the χ^2^ or Fisher exact test, as suitable. Comparisons among the etiology groups, as categorized by the ILAE,^10^ were performed with the χ^2^ test for proportions and the Kruskal-Wallis test for continuous variables. The level of significance for univariable analyses was set at *p* ≤ 0.005 after Bonferroni correction for multiple comparisons.

For multivariable models, relative risks (RRs) of death were estimated by Poisson regression with robust error variance^24^ for all ILAE etiology groups and specific etiologies that differed between survivors and nonsurvivors in univariable comparisons. Therefore, each etiology group or specific etiology was entered as a binary categorical variable and compared with all other etiologies combined. Because every ILAE etiology is a nominal, not ordinal, variable, we fit a series of models, each entering 1 etiology as a binary exposure (i.e., specific etiology group vs all other etiology groups), to estimate adjusted risk ratios for that etiology without imposing any ordering. These models were adjusted for potential confounders (i.e., baseline variables other than etiologies that were selected a priori based on clinical plausibility and previous evidence), such as age, NCSE with coma, and CCI, as well as for the institution. NCSE with coma can be considered as both a confounder and a mediator in the context of our study. NCSE with coma was included into the multivariable model to estimate the direct effect of etiology on outcome. We corrected for potential differences between the 2 sites by including the centers into the models. This is required to isolate the effects of the covariables of interest and to improve the validity and generalizability of results, which may be hampered by unmeasured center-level factors. RRs were used to avoid an overestimation of associations in contrast to odds ratios.^25^ Unlike RRs, odds ratios compare odds rather than probabilities and thus inflate effect sizes as event frequencies rise. Given that the examined outcomes, mortality and recovery to premorbid neurologic function, were likely frequent, logistic regression would have exaggerated associations between predictors and outcomes. As shown,^25^ odds ratios can “overstate” true risk ratios in such contexts. Therefore, we applied Poisson regression with robust error variance to directly estimate RRs, as recommended elsewhere^24^ and in line with STROBE guidelines.^12^ Sensitivity analyses were performed in the subgroup of patients with RSE and in the subgroup of patients with NCSE with coma. The level of significance for univariable analyses was set at *p* ≤ 0.002 after Bonferroni correction for multiple comparisons^26,27^ in line with the following approach: we first performed 28 univariable comparisons in search for variables to be included in the models (i.e., 2 demographics, 5 ILAE SE etiology groups, 15 specific etiologies, 3 main SE type groups, the STESS, SE duration, and the Charlson Comorbidity Index) present at SE onset and calculated a significance threshold *p* value of 0.0018. These analyses were followed by 21 multivariable models (i.e., 7 models for 3 outcomes each), resulting in an adjusted *p* value level of 0.0024. Two-tailed tests were applied throughout.

Deviance and Pearson goodness-of-fit tests were performed for all models to check adequate model fit, indicated by insignificant *p* values. Statistical analysis was performed with STATA 16.1 (Stata Corp., College Station, TX).

### Data Availability

Individual deidentified participant data that underlie the results reported in this article will be shared. The deidentified data sets generated during and/or analyzed for this study will be made available. This includes individual participant data that were used to generate the results in the published article. The data will be made available beginning after article publication and will be accessible for a period of 5 years. Data will be shared on reasonable request to qualified researchers. Requests must be made directly to the corresponding author and must include a scientifically sound proposal for analysis. Access will be granted only to certified investigators (e.g., affiliated with an academic or research institution) who sign a data use agreement obligating them to (1) use the data only for the specified research proposal; (2) not attempt to re-identify any participants; and (3) secure the data using appropriate safeguards. Data will be provided through a secure electronic method after request approval.

## Results

**Figure 1:**
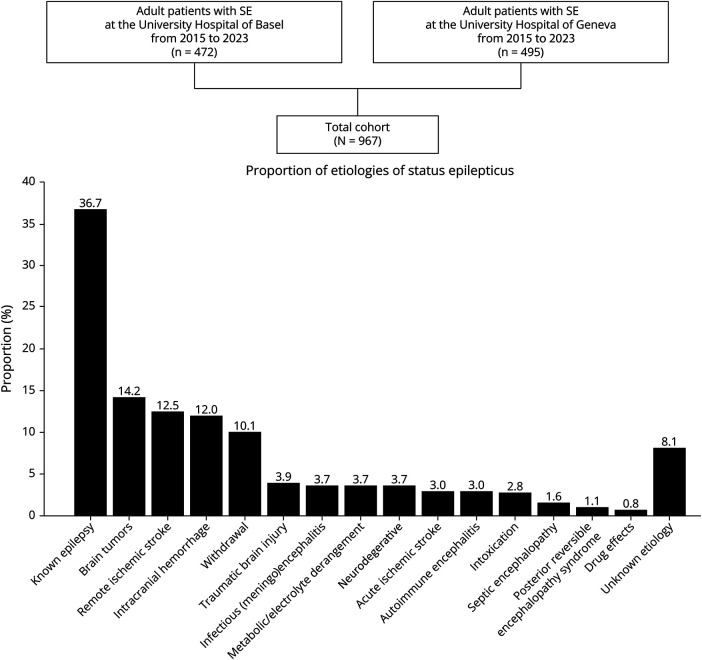
Flowchart SE = status epilepticus.

**Figure 2:**
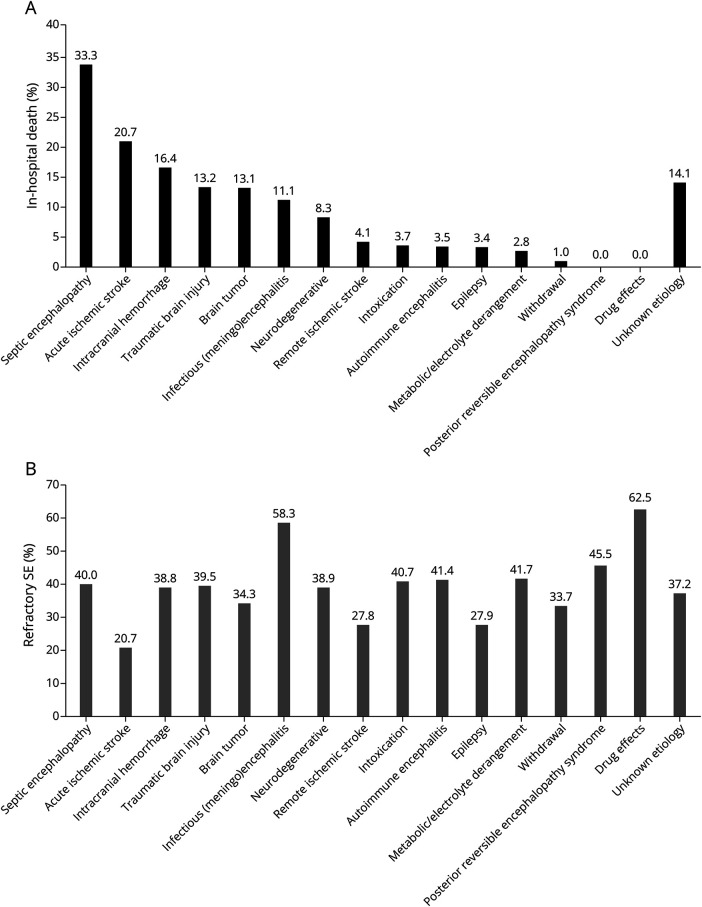
(A) Mortality and (B) Proportion of Refractory SE Among Different Etiologies SE = status epilepticus.

**Figure 3:**
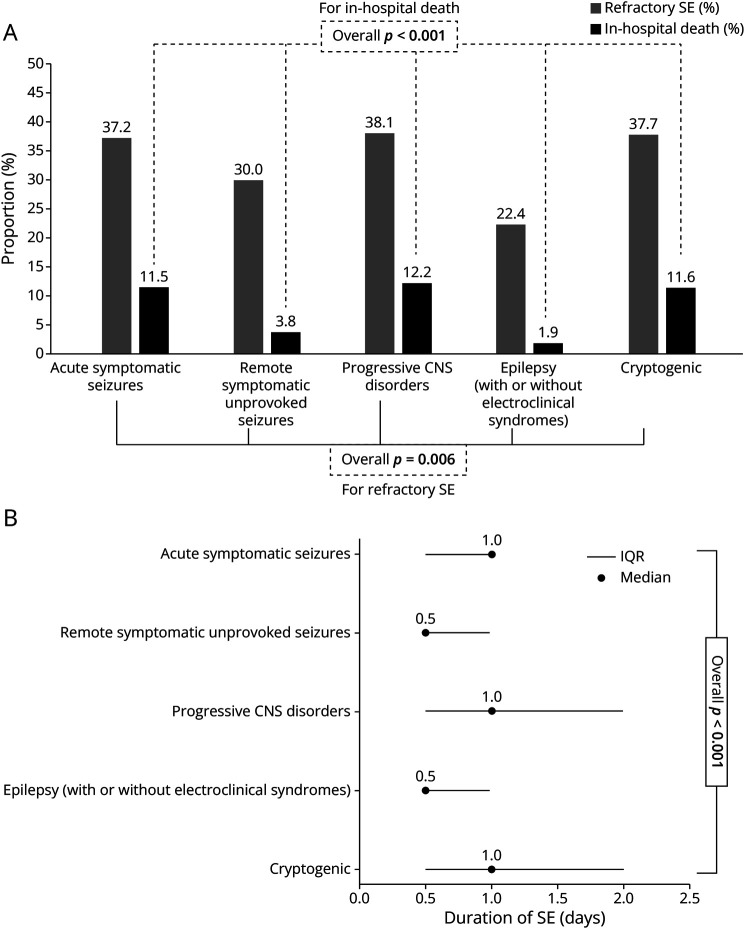
(A) Mortality, Proportion of Refractory SE, and (B) Duration of SE in Different Etiology Groups Categorized According to the ILAE ILAE = International League Against Epilepsy; SE = status epilepticus. Bold font indicates statistical significance.

In total, 967 adult patients with SE were identified. Figure 1 presents the flowchart and distribution of SE etiologies, with the most common being known and uncontrolled epilepsy (36.7%). Demographics, clinical and treatment characteristics, and outcomes are summarized in Table 1. Figure 2 presents the results regarding mortality (Figure 2A) and proportion of refractory SE (Figure 2B). Univariable comparisons of demographics, clinical characteristics, and outcomes between the 2 medical centers are presented in eTable 1. SE was terminated in 95%, with 48.5% recovering to premorbid neurologic function. The overall in-hospital mortality was 7.9% and 13.9% at 30-day follow-up, with loss to follow-up in 27% (eTable 1). Figure 3 shows in-hospital mortality by the ILAE etiology categories, along with the proportions of RSE (Figure 3A) and median SE duration (Figure 3B); all differed significantly across these groups in univariable analyses. eTable 2 presents specific etiologies of SE by ILAE categories, and eTable 3 presents the proportions of etiologies among the specific SE types.

**Table 1:** Demographics, Clinical Characteristics, Treatment, and Outcomes

Demographics and clinical characteristics	Total cohort (n = 967)
Demographics	
Age, y, median (IQR)	67 (54–78)
Female, n (%)	450 (46.5)
SE etiology grouped according to the ILAE^10^, n (%)	
Acute symptomatic seizures	331 (34.2)
Remote symptomatic unprovoked seizures	267 (27.6)
Progressive CNS disorders	139 (14.4)
Epilepsy without additional triggers (with or without underlying electroclinical syndrome)	161 (16.7)
Cryptogenic	69 (7.1)
SE type, n (%)	
SE with prominent motor symptoms	569 (58.8)
SE with motor symptoms (convulsive)	395 (40.8)
SE with motor symptoms (myoclonic)	174 (18.0)
SE without prominent motor symptoms	398 (41.2)
NCSE with coma	115 (11.9)
NCSE without coma	283 (29.3)
Generalized NCSE without coma (i.e., atypical absence status)	3 (0.3)
Focal NCSE without coma (conscious)	65 (6.7)
Focal NCSE without coma (with altered consciousness)	215 (22.2)
Illness severity, median (IQR)	
STESS	3 (2–4)
SE duration, d	0.5 (0.5–1)
CCI	4 (2–6)
Treatment characteristics	
In-hospital stay, d, median (IQR)	10 (5–18)
ICU treatment, n (%)	527 (54.5)
ICU stay, d, median (IQR)	3 (2–8)
Duration of mechanical ventilation of intubated ICU patients, d, median (IQR)	2 (2–7)
No. of nonanesthetic antiseizure drugs, median (IQR)	2 (2–3)
Patients with continuous anesthetic drugs, n (%)	313 (32.4)
Complications during SE, n (%)	
Infections/sepsis	141 (14.6)
Arterial hypotension requiring vasopressors	138 (14.3)
Organ failure	86 (8.9)
WLST, n (%)	
Withdrawal during hospital stay	115 (11.9)
Withdrawal during SE	45 (4.7)
Withdrawal after SE termination	70 (7.2)
Withdrawal due to missing additional treatment options	5 (0.5)
Withdrawal due to presumed poor prognosis	56 (5.8)
Withdrawal according to patients' advanced directives or presumed patient will	54 (5.6)
Withdrawal in ICU patients	60 (6.2) (11.4 of ICU patients)
Outcomes, n (%)	
Persistent seizure termination	919 (95.0)
Return to premorbid neurologic function at discharge	469 (48.5)
In-hospital death	76 (7.9)
30-d mortality (262 patients with loss to follow-up)	98 (13.9)

Abbreviations: CCI = Charlson Comorbidity Index (range 0–37)^17^; ICU = intensive care unit; ILAE = International League Against Epilepsy^10,11^; IQR = interquartile range; NCSE = nonconvulsive status epilepticus; SE = status epilepticus; STESS = Status Epilepticus Severity Score (range 0–6)^15,16^; WLST = withdrawal of life-sustaining treatment.

### Univariable Comparisons Between Survivors and Nonsurvivors

Table 2 presents univariable comparisons of demographics, clinical characteristics, and treatment measures between survivors and nonsurvivors. After correcting for multiple comparisons, nonsurvivors were older and had SE from intracranial hemorrhages more frequently, whereas known and uncontrolled epilepsy was more frequent in survivors. The median Glasgow Coma Score at SE onset in patients with intracranial hemorrhages was 7 (IQR 3–9). SE types also differed: SE with motor symptoms was more common in survivors, whereas NCSE with coma was more frequent in nonsurvivors. Illness severity differed significantly between survivors and nonsurvivors, with a higher STESS, CCI, and longer SE duration in nonsurvivors (Table 2). Treatment differences included more frequent ICU admissions, longer mechanical ventilation, and prolonged ICU stay. Complications during SE were more frequently observed in nonsurvivors, with infections occurring at a higher rate (13.6% in survivors vs 26.3% in nonsurvivors, *p* = 0.003; Table 2).

**Table 2:** Univariable Comparisons of Demographics and Clinical Characteristics Between Survivors and Nonsurvivors

Demographics and clinical characteristics	Survivors (n = 891)	Nonsurvivors (n = 76)	*p* Value^a^
Demographics			
Age, y, median (IQR)	67 (53–78)	74 (64–83)	<0.001^c^
Female, n (%)	413 (46.4)	37 (48.7)	0.696
SE etiology grouped according to the ILAE, n (%)			
Acute symptomatic seizures	293 (32.9)	38 (50.0)	<0.001^c^
Remote symptomatic unprovoked seizures	257 (28.8)	10 (13.2)	
Progressive CNS disorders	122 (13.7)	17 (22.4)	
Epilepsy without additional triggers (with or without underlying electroclinical syndrome)	158 (17.7)	3 (4.0)	
Cryptogenic	61 (6.8)	8 (10.5)	
SE etiology (non–mutually exclusive), n (%)			<0.001^b^^,^^c^
Known epilepsy	343 (38.5)	12 (15.8)	<0.001^c^
Brain tumors	119 (13.4)	18 (23.7)	0.013
Remote ischemic stroke	116 (13.0)	5 (6.6)	0.146
Intracranial hemorrhage	97 (10.9)	19 (25.0)	<0.001^c^
Withdrawal	97 (10.9)	1 (1.3)	0.006
Traumatic brain injury	33 (3.7)	5 (6.6)	
Infectious (meningo)encephalitis	32 (3.6)	4 (5.3)	
Metabolic/electrolyte derangement	35 (3.9)	1 (1.3)	
Neurodegenerative	33 (3.7)	3 (4.0)	
Acute ischemic stroke	23 (2.6)	6 (7.9)	
Autoimmune encephalitis	28 (3.1)	1 (1.3)	
Intoxication	26 (2.9)	1 (1.3)	
Septic encephalopathy	10 (1.1)	5 (6.6)	
Posterior reversible encephalopathy syndrome	11 (1.2)	0 (0.0)	
Drug effects	8 (0.9)	0 (0.0)	
SE type, n (%)			
SE with prominent motor symptoms	542 (60.8)	27 (35.5)	<0.001^c^
SE with motor symptoms (convulsive)	382 (42.9)	13 (17.1)	
SE with motor symptoms (myoclonic)	160 (18.0)	14 (18.4)	
SE without prominent motor symptoms	349 (39.2)	49 (64.5)	<0.001^c^
NCSE with coma	86 (9.7)	29 (38.2)	<0.001^c^
NCSE without coma	263 (29.5)	20 (26.3)	0.556
Generalized NCSE without coma (i.e., atypical absence status)	3 (0.3)	0 (0.0)	
Focal NCSE without coma (conscious)	62 (7.0)	3 (4.0)	
Focal NCSE without coma (with altered consciousness)	198 (22.2)	17 (22.4)	
Illness severity, median (IQR)			
STESS	3 (2–4)	4 (3–5)	<0.001^c^
SE duration, d	0.5 (0.5–1)	2 (1–6)	<0.001^c^
CCI	4 (2–6)	5 (4–7)	<0.001^c^
Treatment characteristics			
In-hospital stay, d, median (IQR)	10 (5–18)	10 (6–21)	0.321
ICU treatment, n (%)	489 (54.9)	38 (50.0)	0.412
ICU stay, d, median (IQR)	3 (2–7)	8 (3–17)	<0.001^c^
Duration of mechanical ventilation of intubated ICU patients, d, median (IQR)	2 (0.5–6)	10 (5–15)	<0.001^c^
No. of nonanesthetic antiseizure drugs, median (IQR)	2 (2–3)	3 (2–4)	0.040
Patients with continuous anesthetic drugs, n (%)	292 (32.8)	21 (27.6)	0.358
Complications during SE, n (%)			
Infections/sepsis	121 (13.6)	20 (26.3)	0.003
Arterial hypotension requiring vasopressors	121 (13.6)	17 (22.4)	0.036
Organ failure	77 (8.6)	9 (11.8)	0.347
WLST, n (%)			
Withdrawal during hospital stay	55 (6.2)	60 (79.0)	<0.001^c^
Withdrawal during SE	17 (1.9)	28 (36.9)	<0.001^c^
Withdrawal after SE termination	38 (4.3)	32 (42.1)	<0.001^c^
Withdrawal in ICU patients	55 (6.2)	60 (79.0)	<0.001^c^

Abbreviations: CCI = Charlson Comorbidity Index (range 0–37)^17^; ICU = intensive care unit; ILAE = International League Against Epilepsy^10,11^; IQR = interquartile range; NCSE = nonconvulsive status epilepticus; SE = status epilepticus; STESS = Status Epilepticus Severity Score (range 0–6)^15,16^; WLST = withdrawal of life-sustaining treatment.

a Level of significance set at *p* ≤ 0.002 after Bonferroni correction for multiple comparisons.

b Overall *p* value across all etiologies.

c Values indicate statistical significance.

Analyses regarding WLST during SE revealed that, among patients without WLST specified in their advance directives, the median duration from SE onset to withdrawal was 3 days (IQR 1–9). Analyses regarding the number of patients with WLST revealed that, of all patients with withdrawal (n = 115), 52.2% (n = 60) died during their hospital stay and 25.2% (n = 29) were transferred to hospices. Of the remaining 22.6% (n = 26), 18 were transferred to another hospital and 8 were discharged home. Of the 61 patients with WLST and no directives, 16 (26.2%) had SE with prominent motor symptoms (9 [14.8%] convulsive; 7 [11.5%] myoclonic) and 44 (72.1%) had SE without prominent motor symptoms (23 [37.7%] NCSE with coma; 21 [34.4%] NCSE without coma). These 61 patients had a median age of 74 (IQR 64–82) years, a high median Charlson Comorbidity Index (5, IQR 3–7), and a median STESS of 5 (IQR 3–5). Ten patients (16.4%) developed organ failure, and another 10 (16.4%) had metastatic tumors.

### Multivariable Regression Analyses

Table 3 presents multivariable regression analyses to identify factors available early during SE and independently associated with primary and secondary outcomes. These models included the ILAE etiology groups, specific etiologies, and baseline variables differing between survivors and nonsurvivors in univariable comparisons. Each reported risk ratio compared the indicated etiology with all other etiologies combined (separate model per etiology), adjusting for age, NCSE with coma, Charlson Comorbidity Index, and center. With the level of significance at *p* ≤ 0.002, no SE etiology or etiology group was associated with RRs of mortality independent of confounders, except remote symptomatic unprovoked SE, which was associated with lower 30-day mortality. However, etiology was independently associated with survival with full recovery (i.e., return to premorbid neurologic functions at discharge), with intracranial hemorrhage and acute symptomatic seizures decreasing the likelihood, while remote symptomatic unprovoked seizures or seizures of unknown cause increased it (Table 3). The significance of these associated RRs did not markedly change after the exclusion of patients with WLST by following patients' directives (Table 4) or restriction of our analyses to patients treated with or without intensive care (eTable 4).

**Table 3:** Multivariable Models for the RRs of Etiologies for Specific Primary and Secondary Outcomes (n = 967)

Etiologies^a^	Primary outcome			Secondary outcomes					
	In-hospital death			30-d mortality (27% lost to follow-up)			Survival with return to premorbid neurologic function at discharge		
	RR	95% CI	*p* Value^b^^,^^c^	RR	95% CI	*p* Value^b^^,^^c^	RR	95% CI	*p* Value^b^^,^^c^
SE etiologies grouped according to the ILAE									
Acute symptomatic seizures	1.67	1.09–2.54	0.017	1.50	1.05–2.13	0.026	0.71	0.60–0.83	<0.001^d^
Age	1.02	1.00–1.03	0.045	1.03	1.01–1.05	<0.001^d^	0.99	0.99–1.00	0.032
NCSE with coma	5.12	3.30–7.95	<0.001^d^	3.87	2.70–5.55	<0.001^d^	0.51	0.36–0.70	<0.001^d^
Charlson Comorbidity Index	1.13	1.04–1.22	0.004	1.10	1.02–1.18	0.010	0.95	0.92–0.98	0.004
Remote symptomatic unprovoked seizures	0.42	0.22–0.83	0.012	0.42	0.23–0.72	0.002^d^	1.21	1.06–1.38	0.004
Age	1.02	1.00–1.03	0.025	1.03	1.02–1.05	<0.001^d^	0.99	0.99–1.00	<0.001^d^
NCSE with coma	4.96	3.23–7.61	<0.001^d^	3.62	2.54–5.16	<0.001^d^	0.48	0.35–0.67	<0.001^d^
Charlson Comorbidity Index	1.11	1.03–1.21	0.008	1.09	1.02–1.16	0.016	0.95	0.92–0.99	0.006
Progressive CNS disorders	1.35	0.84–2.19	0.127	1.51	1.03–2.23	0.036	0.72	0.57–0.92	0.008
Age	1.02	1.00–1.04	0.023	1.03	1.02–1.05	<0.001^d^	0.99	0.99–1.00	0.010
NCSE with coma	5.53	3.63–8.43	<0.001^d^	4.12	2.93–5.80	<0.001^d^	0.47	0.34–0.66	<0.001^d^
Charlson Comorbidity Index	1.11	1.02–1.20	0.015	1.08	1.01–1.15	0.031	0.96	0.93–1.00	0.028
Epilepsy without additional triggers (with or without underlying electroclinical syndrome)	0.37	0.12–1.20	0.098	0.62	0.40–0.96	0.003	1.52	1.33–1.74	<0.001^d^
Age	1.02	1.00–1.03	0.051	1.02	1.01–1.03	<0.001^d^	1.00	1.00–1.01	0.035
NCSE with coma	5.21	3.41–8.00	<0.001^d^	2.18	1.72–2.78	<0.001^d^	0.50	0.36–0.70	<0.001^d^
Charlson Comorbidity Index	1.11	1.03–1.21	0.009	1.09	1.04–1.14	<0.001^d^	0.96	0.93–0.99	0.020
Cryptogenic	1.12	0.54–2.35	0.756	0.90	0.61–1.33	0.598	1.08	0.84–1.39	0.560
Age	1.02	0.99–1.03	0.060	1.02	1.01–1.03	<0.001^d^	0.99	0.99–1.00	0.026
NCSE with coma	5.61	3.66–8.59	<0.001^d^	2.32	1.83–2.95	<0.001^d^	0.47	0.34–0.65	<0.001^d^
Charlson Comorbidity Index	1.12	1.03–1.22	0.006	1.09	1.05–1.14	<0.001^d^	0.95	0.92–0.99	0.005
Specific etiologies of SE									
Known epilepsy	0.49	0.26–0.92	0.027	0.51	0.30–0.88	0.014	1.40	1.23–1.60	<0.001^d^
Age	1.02	1.00–1.03	0.067	1.03	1.01–1.04	0.001^d^	1.00	0.99–1.00	0.057
NCSE with coma	4.91	3.18–7.58	<0.001^d^	3.73	2.63–5.28	<0.001^d^	0.51	0.36–0.71	<0.001^d^
Charlson Comorbidity Index	1.10	1.02–1.19	0.012	1.08	1.01–1.16	0.019	0.96	0.93–1.00	0.028
Intracranial hemorrhage	1.71	1.04–2.83	0.036	1.57	1.04–2.36	0.031	0.49	0.35–0.67	<0.001^d^
Age	1.02	1.00–1.03	0.046	1.03	1.01–1.04	<0.001^d^	1.00	0.99–1.00	0.064
NCSE with coma	5.09	3.26–7.93	<0.001^d^	3.99	2.82–5.66	<0.001^d^	0.51	0.37–0.71	<0.001^d^
Charlson Comorbidity Index	1.12	1.03–1.22	0.006	1.10	1.02–1.18	0.009	0.95	0.92–0.98	0.004

Abbreviations: ILAE = International League Against Epilepsy^10,11^; NCSE = nonconvulsive status epilepticus; RR = relative risk; SE = status epilepticus.

Deviance and Pearson goodness-of-fit tests for all models revealed insignificant *p* values indicating adequate model fit.

a Each etiology group is compared with all other etiologies as reference and is modeled separately.

b All models also adjusted for medical center.

c Level of significance set at *p* ≤ 0.002 after Bonferroni correction for multiple comparisons.

d Values indicate statistical significance.

**Table 4:** Multivariable Models for the RRs of Etiologies for Specific Primary and Secondary Outcomes After Exclusion of Patients With Withdrawal of Life-Sustaining Treatment After Patients' Directives or Presumed Patients' Will (n = 913)

Etiologies^a^	Primary outcome			Secondary outcomes					
	In-hospital death			30-d mortality (0.8% lost to follow-up)			Survival with return to premorbid neurologic function at discharge		
	RR	95% CI	*p* Value^b,c^	RR	95% CI	*p* Value^b,c^	RR	95% CI	*p* Value^b,c^
SE etiology grouped according to the ILAE									
Acute symptomatic seizures	1.67	0.98–2.84	0.062	1.52	1.00–2.36	0.050	0.71	0.61–0.83	<0.001^d^
Remote symptomatic unprovoked seizures	0.35	0.15–0.85	0.020	0.37	0.19–0.74	0.005	1.19	1.05–1.35	0.008
Progressive CNS disorders	1.58	0.86–2.93	0.143	1.91	1.18–3.11	0.009	0.71	0.56–0.90	0.005
Epilepsy without additional triggers (with or without underlying electroclinical syndrome)	0.33	0.08–1.39	0.131	0.62	0.39–0.98	0.041	1.53	1.34–1.75	<0.001^d^
Cryptogenic	1.27	0.53–3.04	0.597	0.82	0.51–1.32	0.412	1.12	0.87–1.43	0.371
Specific underlying etiology of SE									
Known epilepsy	0.46	0.22–0.98	0.046	0.52	0.28–1.00	0.039	1.40	1.23–1.59	<0.001^d^
Intracranial hemorrhage	1.91	1.01–3.63	0.048	1.86	1.12–3.06	0.016	0.49	0.36–0.68	<0.001^d^

Abbreviations: ILAE = International League Against Epilepsy^10,11^; RR = relative risk; SE = status epilepticus.

Deviance and Pearson goodness-of-fit tests for all models revealed insignificant *p* values indicating adequate model fit.

a Each etiology group is compared with all other etiologies as reference and is modeled separately.

^b^All models adjusted for medical center, age, nonconvulsive SE with coma, and Charlson Comorbidity Index.

c Level of significance set at *p* ≤ 0.002 after Bonferroni correction for multiple comparisons.

d Values indicate statistical significance.

Overall, NCSE with coma showed the strongest independent association with mortality (both in-hospital and at 30 days) across all SE etiologies (Table 3), and was also associated with a lower likelihood of achieving premorbid neurological function. In line with all multivariable analyses, these comparisons were performed using a separate statistical model for each etiology, comparing it with all other etiologies combined to derive its specific risk ratio.

### Sensitivity Analyses

Sensitivity analyses for patients with RSE are presented in eTable 5. In this subgroup, NCSE with coma remained strongly, independently, and uniquely associated with in-hospital mortality and 30-day mortality, whereas all other variables were no longer associated with these outcomes. Etiology, on the contrary, lost its association with survival with return to premorbid neurologic function at discharge.

In addition, we performed sensitivity analysis for patients with NCSE with coma regarding the association of etiology with the primary outcome (eTable 6). These analyses revealed no associations with primary outcome except for epilepsy without additional triggers with or without electroclinical syndromes, which was inversely associated with in-hospital death (i.e., with no deaths recorded with this etiology group). When NCSE with coma was excluded from the multivariable models, especially the ILAE etiology group, acute symptomatic seizures became significantly associated with an increased RR of in-hospital mortality (RR 2.04, 95% CI 1.33–3.10, *p* = 0.001), whereas remote symptomatic unprovoked seizures were associated with a decreased risk (RR 0.34, 95% CI 0.17–0.65, *p* = 0.001). This attenuation pattern observed after adjustment for NCSE with coma supports the notion that NCSE with coma may partially mediate the relationship between SE etiology and mortality. All sensitivity analysis models were applied for each etiology, comparing it with all other etiologies combined.

## Discussion

This 2-center study investigated the impact of SE etiologies, including those as categorized by the ILAE,^10,11^ on mortality and neurologic recovery while adjusting for potential confounders and accounting for the effect of multiple comparisons and the withdrawal of life-sustaining measures.

Based on a cohort of almost 1,000 patients, our analyses revealed that mortality and the proportion of RSE differed significantly across the etiology groups categorized according to the ILAE (Figure 3). However, after adjusting for potential confounders (i.e., age, NCSE with coma, and CCI), no specific etiology or etiology group was independently associated with short-term mortality, although both remained associated with neurologic recovery (Table 3). SE due to intracranial hemorrhage and acute symptomatic etiologies was associated with a lower chance of survival with full recovery to premorbid neurologic function, as was NCSE with coma. By contrast, known and uncontrolled epilepsy, remote symptomatic epilepsy, and unprovoked/unknown etiology were associated with improved functional outcomes. The findings regarding NCSE with coma and poor outcomes were even more pronounced in patients with RSE while specific etiologies lost their association with survival with return to neurologic function at discharge. The latter may be explained by the emergence of RSE that may counteract the outcome advantages of less severe etiology. Sensitivity analyses revealed inverse associations between epilepsy without additional triggers (with or without underlying electroclinical syndrome) and in-hospital death, with no deaths recorded in this subgroup. The latter suggests that while NCSE with coma is frequently associated with increased mortality, its emergence in the context of epilepsy without additional triggers seems less fatal.

Our study population from 2 academic care centers demonstrates similar clinical characteristics to those in other SE studies, including age,^3,9,28-34^ outcome,^3,6,9,29,31,33-37^ etiologies,^3,7,9,29-31,33,35^ complications,^6,9,32,36^ SE severity,^3,6,9,29,30,33,34,37^ and types of SE.^3,6,9,28,33-35,37^ The observed similarities indicate the potential for generalizability to broader populations with similar socioeconomic characteristics in comparable clinical settings. Our findings further align with previous studies emphasizing the importance of SE etiology in determining clinical outcomes but challenge the notion that etiology is a direct predictor of mortality after adjusting for confounders. Previous research highlighted that SE of acute symptomatic origin, particularly when secondary to conditions such as stroke, traumatic brain injury, or intracranial hemorrhage, carries a higher mortality risk.^1-3,5-8^ However, these earlier studies often lacked standardized etiologic categorization, adjustment for confounding, and correction of multiple comparisons.

The observed negative impact of intracranial hemorrhage and acute symptomatic SE on reduced neurologic recovery aligns with findings from prospective cohorts, indicating that these etiologies frequently lead to brain damage, increased intracranial pressure, and higher rates and duration of RSE, which collectively impair functional recovery.^5,9,28^ Conversely, the higher likelihood of favorable outcomes in patients with known epilepsy or unprovoked SE is supported by previous studies, suggesting that these patients have a larger neurologic reserve and may respond better to treatment.^1,3,16,38,39^

While WLST after 3 days of SE seems short, these 3 days of SE do not include pre-SE ICU treatment, which had a median duration of 6 days. Furthermore, of the 61 patients with WLST and no directives, 26% had SE with prominent motor symptoms (15% convulsive; 12% myoclonic) and 72% had SE without prominent motor symptoms (38% NCSE with coma; 34% NCSE without coma). However, they were also older, were burdened with comorbidities, and had a poor outcome prediction as indicated by their STESS, accompanied by development of organ failure in a subset. Which of these aspects may have driven the decision to withdraw life-sustaining treatment cannot be reliably determined in retrospect.

Although it seems plausible that WLST alters patient outcomes, this did not significantly affect the association between etiology and outcomes (Table 4). This provides an important methodological insight because WLST has been recognized as an underacknowledged confounder in SE studies, often leading to overestimation of mortality in specific etiologies.^5^ By addressing this issue, our study enhances the reliability of its conclusions regarding the role of etiology in neurologic recovery. WLST may result from nihilism of the treating physicians. However, our data indicate that, in 44% of patients with WLST, withdrawal was performed by following the instructions of the patients' advance directives. Among the remaining 56% of patients without such advance directives, the median duration from SE onset to withdrawal during SE was 3 days (IQR 1–9). The latter suggests prolonged efforts in these cases to terminate SE.

In addition, our study shows that NCSE with coma is the strongest predictor of outcome, being independently linked to increased mortality and decreased chances of neurologic recovery throughout all our analyses. Of interest, although intracranial hemorrhage was frequently diagnosed (22.6%) among patients with NCSE with coma, inclusion of both variables in the same multivariable models showed that each was independently associated with reduced survival with return of neurological function; however, with respect to mortality, NCSE with coma remained the only significant association. The findings are consistent with previous literature, as several studies have demonstrated that NCSE in critically ill patients is associated with prolonged ICU stays, increased complication rates, and higher mortality compared with convulsive SE.^6,34,37,40,41^ Moreover, the results of our sensitivity analyses further suggest that NCSE with coma may act as a mediator in the relationship between SE etiology and outcome. Specifically, when NCSE with coma was excluded from the multivariable models, the ILAE etiology group “acute symptomatic seizures” became significantly associated with an increased RR of in-hospital mortality, whereas “remote symptomatic unprovoked seizures” were associated with a decreased risk.

While etiology is an integral component of the Epidemiology-based Mortality Score in SE (EMSE),^42^ the more frequently used STESS demonstrates a reduced emphasis on etiology because it solely relies on a preexisting epilepsy diagnosis.^15,16,43^ The latter suggests that, relative to other clinical variables, the specific underlying cause of SE may contribute less to outcome prediction. Current, yet limited, data indicate that mortality prediction models incorporating weighted etiologic factors, such as the EMSE, may exhibit improved accuracy.

In line with our findings, NCSE is possibly the most crucial component of the STESS, contributing to higher scores and thereby indicating worse prognosis.^15,16^ Of interest, modified STESS versions have been proposed, for example, by incorporating the baseline modified Rankin Scale^44^ or by replacing age with hypoalbuminemia^45^; however, external validation of these modified systems is lacking, and none of these versions replaced or modified NCSE, the latter remaining the strongest variable related to hospital mortality.

The pathophysiologic basis for this poor prognosis likely involves delayed diagnosis of initially unwitnessed or unrecognized SE,^4,46^ persistent seizures leading to secondary neuronal injury, and consecutively higher RSE incidence rates. The latter may represent an important indicator, because RSE and its duration are associated with increased mortality.^3,5^ In line with these studies, RSE was also more frequent among nonsurvivors in our cohort.

The fact that NCSE with coma is the main predictor of outcome in the SE population highlights the need for cEEG monitoring of patients at risk of seizures and SE, in the ICU setting, and for a prompt and tailored management when NCSE is detected, to prevent the deleterious consequences of ongoing epileptic activity on neuronal networks and tissues. On the contrary, it cannot be excluded that “overtreatment” in NCSE may lead to or sustain coma, worsening brain function. Therefore, integrating cEEG with neurologic expertise in the critical care setting seems to be the best approach.^47,48^

As outlined above, our study's strengths include the large and representative cohort of adult patients with SE categorized according to the ILAE, the adjustment for potential confounders, and consideration of WLSTs. As discussed, the clinical characteristics of our population align closely with those reported in other SE cohorts, supporting the generalizability of our findings to other SE populations in similar clinical settings. SE management and treatment were closely monitored by epileptologists and neurocritical care specialists at both centers through daily assessments. This ensured a high degree of adherence to international guidelines in diagnostic and therapeutic procedures. Another strength is the correction for multiple comparisons and the goodness-of-fit tests to assure adequate model fit for all regression models.

However, there are limitations to be addressed, such as the restriction to 2 centers and the observational design limiting the generalizability of our results.

Another limitation is the category summarizing patients with SE emerging from known epilepsy without identifiable additional triggers and patients with electroclinical syndromes. This summarizing categorization was necessary because, in retrospect, we were unable to identify patients with specific electroclinical syndromes and to delineate them from patients with SE emerging from epilepsy without additional triggers. Similarly, although refractoriness was captured, we could not reliably identify new-onset RSE (NORSE) because its definition requires both new-onset refractory SE and detailed etiologic investigations, which were not available; some patients within the cryptogenic and acute symptomatic groups may, therefore, overlap with NORSE.

The loss to follow-up in 27% of patients represents a potential source of selection bias regarding our 30-day mortality analysis. Another limitation is the approximation and, therefore, potential underestimation of the SE duration, especially with unwitnessed onset, mainly in the case of NCSE.^4^ However, because SE duration was not a study end point, this has little impact on our result. Finally, owing to the retrospective study design, the number of patients with super-refractory SE could not be determined because we were not able to clearly differentiate patients in whom treatment was escalated after the establishment of anesthesia due to RSE from patients in whom anesthesia was escalated or changed for other reasons (e.g., concerns that SE might rebound or anesthetic-related side effects).

Our study challenges the notion that SE etiology is a direct predictor of mortality when adjusting for confounders and accounting for WLST. Our data, however, underscore that etiology is a critical determinant of neurologic recovery. In contrast to etiology, the consistent association of NCSE with coma with increased risks of short-term and 30-day mortality independent of potential confounders emphasizes the need for heightened clinical vigilance, cEEG monitoring, and prompt and tailored management of unconscious patients with NCSE. These insights contribute to growing evidence highlighting the role of structured etiologic classification in improving prognostic assessment and guiding therapeutic strategies in SE management.

## Glossary

CCI: Charlson Comorbidity Index
cEEG: continuous EEG
EMSE: Epidemiology-based Mortality Score in SE
ICU: intensive care unit
ILAE: International League Against Epilepsy
IQR: interquartile range
NCSE: nonconvulsive SE
NORSE: new-onset RSE
RSE: refractory SE
SE: status epilepticus
STESS: Status Epilepticus Severity Score
STROBE: Strengthening the Reporting of Observational Studies in Epidemiology
WLST: withdrawal of life-sustaining treatment
